# Fine-Mapping and Functional Characterization of *BjMYB113* in Controlling Purple Leaf Coloration in *Brassica juncea*

**DOI:** 10.3390/plants15213463

**Published:** 2026-11-06

**Authors:** Zhihuan Ge, Mengyi Yu, Yinge Xie, Haoran Wang, Shaojun Ma, Yijian Liu, Shanshan Zhou, Xueliang Sun, Feifei Yu, Mingxia Wang

**Affiliations:** 1 Institute of Vegetables, Anhui Academy of Agricultural Sciences, Hefei 230011, China; gzh72689@163.com (Z.G.); xieyinge0518@126.com (Y.X.);; 2 Anhui Provincial Key Laboratory for Germplasm Resources Creation and High-Efficiency Cultivation of Horticultural Crops, Hefei 230011, China; 3 Key Laboratory of Horticultural Crop Germplasm Innovation and Utilization (Co-Construction by Ministry and Province), Hefei 230011, China; 4 Institute of Horticulture, Anhui Academy of Agricultural Sciences, Hefei 230011, China; 5 Department of Horticulture, Beijing Vocational College of Agriculture, Beijing 102442, China; 6 Jinzhai County Agricultural Industry Development Center, Jinzhai 237300, China

**Keywords:** flavonoid biosynthesis, anthocyanin, gene mapping, *BjMYB113*

## Abstract

The purple leaves of *Brassica juncea* are abundant in anthocyanins that confer stress tolerance, nutritional benefits, and ornamental value. In this study, we constructed F_2_ and BC_1_ segregating populations by crossing the purple leaf inbred line (ZY) with the green leaf inbred line (LY) to investigate the genetic basis of leaf pigmentation. Bulked segregant analysis sequencing (BSA-Seq) and map-based cloning localized the *BjZY* locus to a 162.08 kb interval on chromosome BB_Chr05. Based on sequence alignment and homology analysis, an R2R3 MYB transcription factor, *BjMYB113* (*BjuVB05G51160*), was identified as a strong candidate gene, with 3 SNPs and 25 bp insert variants in the promoter region. The expression of *BjMYB113* in purple leaves was significantly higher than that in green leaves, leading to anthocyanin biosynthesis and accumulation. The transient expression of *BjMYB113* in tobacco leaf confirmed that *BjMYB113* positively regulates anthocyanin biosynthesis. RNA-seq analysis revealed that *BjMYB113* was involved in flavonoid and anthocyanin biosynthesis pathways and regulated the expression of anthocyanin-related genes, such as *BjCHS*, *BjDFR*, and *BjANS*. Yeast one-hybrid assays showed that *BjMYB113* activated the expression of *BjDFR* by directly binding to its promoter. These results provide further understanding of the genetics of purple leaf and potential applications for marker-assisted breeding in *B. juncea*.

## 1. Introduction

*Brassica juncea* (L.) Czern., (AABB, 2*n* = 36), commonly called mustard, is an allopolyploid vegetable cultivated worldwide with high economic and nutritional value. Mustard was formed by hybridization between the diploid progenitors *B. rapa* (AA) and *B. nigra* (BB) [1,2]. This cultivated species formed various different varieties with considerable morphological variation utilized for their seeds, leaves, roots, and stems as edible organs. Some varieties are enriched in anthocyanins in the leaf and display a purple leaf color, endowing them with both ornamental value and nutritional quality.

Anthocyanins are water-soluble vacuolar pigments that produce red, purple, and blue coloration in plant tissues. They protect plants from UV-B radiation, cold, drought, and pathogen attack, and serve as dietary antioxidants with documented benefits for human health [3,4,5,6]. The anthocyanin biosynthetic pathway is among the best characterized secondary metabolic pathways in plants. It proceeds through conserved enzymatic steps catalyzed by chalcone synthase (*CHS*), chalcone isomerase (*CHI*), flavanone 3-hydroxylase (*F3H*), flavonoid 3′-hydroxylase (*F3′H*), dihydroflavonol 4-reductase (*DFR*), anthocyanidin synthase (*ANS*), and UDP-glucose:flavonoid glucosyltransferase (*UFGT*) [7,8]. The transcription of these structural genes is controlled by the MBW (MYB-bHLH-WD40) ternary complex, a regulatory module conserved across angiosperms [9,10,11]. Within this complex, R2R3-MYB transcription factors determine target gene specificity and act as primary activators of anthocyanin genes. The bHLH partners (*TT8*, *GL3*, and *EGL3*) and the WD-repeat protein TTG1 stabilize the complex and enhance trans-activation [12,13].

In Arabidopsis thaliana, four R2R3-MYB genes constitute the S6 subgroup defined by the conserved KPRPR[S/T]F motif in the C-terminal domain, including *MYB75* (*PAP1*), *MYB90* (*PAP2*), *MYB113*, and *MYB114* [14,15]. These MYB transcription factors function as redundant positive regulators of anthocyanin accumulation. Ectopic expression of *MYB75* or *MYB113* suffices to induce anthocyanin production in normally unpigmented tissues, and both directly activate promoters of biosynthetic genes such as *DFR*, *ANS*, and *UFGT* [16,17,18]. The S6 subgroup is also subject to negative feedback. The R3-MYB repressor *MYBL2* competes with R2R3-MYB activators for bHLH binding and suppresses the expression of *PAP1*/*PAP2*, thereby fine-tuning anthocyanin levels under light and sucrose signals [15,19,20]. This balance between activator and repressor extends the canonical MBW model and is increasingly recognized as a key determinant of tissue-specific anthocyanin patterning [21,22]. Homologous S6 regulators have been functionally characterized in diverse species. For example, the *Petunia anthocyanin2* (*AN2*) gene encodes a petal-specific MYB transcription factor that influences flower pigmentation through regulating anthocyanin biosynthesis and vacuolar pH in petal epidermal cells [23,24,25]. In grape (*Vitis vinifera*), *VvMYBA1* controls skin anthocyanin biosynthesis by directly activating *UFGT*, and loss-of-function mutations in *VvMYBA1* and the adjacent *VvMYBA2* were shown to account for the origin of white grape cultivars [26]. In banana (*Musa acuminata*), five R2R3-MYB genes (*MusaUP1*, *MusaUP2*, *MbaMA2*, *MusaMA4*, and *MusaMA8*) differentially synchronize anthocyanin biosynthesis in bract through TT8-dependent/independent interactions and differential affinities to *CHS*, *ANR*, *UFGT*, *FLS*, and *ANS*, thereby directing metabolic flux toward distinct pigment end-products [27].

A systematic survey across 31 Brassicaceae species identified 92 S6 homologs, approximately 70% of which clustered in a single conserved clade, with prominent tandem duplications on chromosomes A07 and C06 in Brassica [28]. In *B. rapa*, the dominant purple-heading trait was controlled by *BrMYB2* on chromosome A07, while *BrMYBL2.1* on the same chromosome functioned as a negative regulator whose loss permits anthocyanin accumulation [29,30]. The CRISPR/Cas9-mediated knockout of *BrDFR* in purple Chinese cabbage redirected flavonoid flux and eliminated anthocyanin accumulation, providing the first loss-of-function proof for a structural gene in *B. rapa* [31]. In *B. oleracea*, *BoPAP1* and the structural gene *BoDFR1* independently controlled purple leaf and ornamental kale phenotypes, validated by map-based cloning and gene knockout [32]. A recent study used haploid induction in combination with CRISPR/Cas-based editing (HI-Edit) to rapidly generate transgene-free cabbage lines with tailored anthocyanin contents, demonstrating the translational potential of these regulatory genes for crop improvement [33]. In allotetraploid *B. napus* (AACC), the overexpression of *BnPAP2* from A07 or *OvPAP2* from *Orychophragmus violaceus* activates anthocyanin genes, whereas *BnbHLH92a* negatively regulates seed anthocyanin content [28,31]. Fine mapping in *B. napus* identified *BnaPAP2.C6a* and *BnaPAP2.A7b* as R2R3-MYB candidates controlling pigment accumulation, and *BnaA07.PAP2* was identified as a key regulator of flower coloration across Brassica crops [34,35]. In *B. juncea*, a 1268 bp insertion in the first intron of *BjMYB113 (previously designated BjPur)* changed the purple leaves to green. In addition, the introgression of *BjMYB113* from *B. juncea* into *B. napus* conferred purple leaves, and the knockout of *BjMYB113* in the introgressed line suppressed anthocyanin accumulation [31].

In this study, we investigated the inheritance of mustard leaf color in F_1_, F_2_, and BC_1_ populations derived from a cross of LY (green) and ZY (purple) lines. BSA-seq, fine mapping, sequence homology analysis and functional analysis delimited the candidate genes, *BjMYB113*, to a 162.08 kb interval on chromosome BB_Chr05. Variants in the promoter of *BjMYB113* are likely associated with the altered leaf coloration. RNA-seq analysis and Y1H assays revealed the molecular mechanisms of *BjMYB113* in controlling purple leaf coloration in *B. juncea*. This research provides new insights for dissecting the genetic mechanism underlying purple leaf coloration in mustard, and may facilitate marker-assisted selection in future breeding.

## 2. Results

### 2.1. Phenotypic Characterization of Purple and Green Leaf Lines

**Figure 1:**
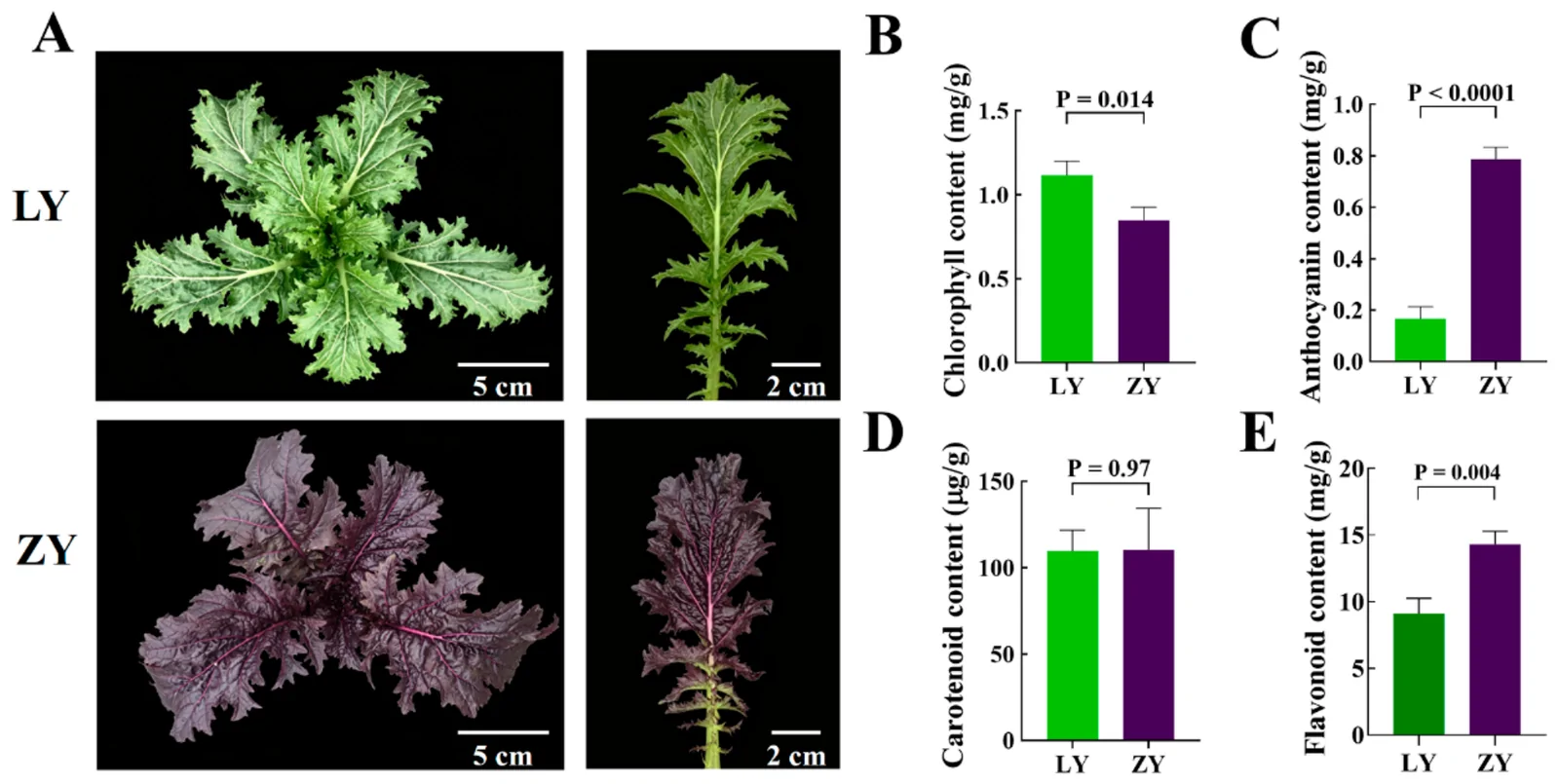
Phenotypic characterization and pigment contents of green leaf (LY) and purple leaf (ZY) lines. (**A**) Leaf phenotypes of LY and ZY lines. (**B**–**E**) Contents of chlorophyll (**B**), anthocyanins (**C**), carotenoids (**D**), and flavonoids (**E**) in LY and ZY leaves. Data are presented as means ± standard errors. Significant differences were determined by Student’s *t*-test.

The purple-leaf line ZY and the green-leaf line LY showed stable and contrasting leaf coloration throughout development. In ZY, the leaves showed purple pigmentation with green petioles, while the LY leaves remained uniformly green at all stages (Figure 1A). No intermediate or chimeric phenotypes were observed in either parent. Four compounds associated with leaf coloration were detected to investigate the basis of the purple pigmentation in ZY leaves. The chlorophyll content of ZY (0.85 mg/g) was significantly lower than that of LY (1.13 mg/g; Figure 1B). The anthocyanin content in ZY leaves was 0.79 mg/g, which was significantly higher than the 0.18 mg/g in LY leaves (Figure 1C). No significant difference in carotenoid content was detected between the ZY and LY leaves (Figure 1D). The flavonoid content of ZY (14.30 mg/g) was significantly different from that of LY (Figure 1E). The accumulation of anthocyanins and flavonoids was positively correlated with the leaf color of ZY.

**Figure 2:**
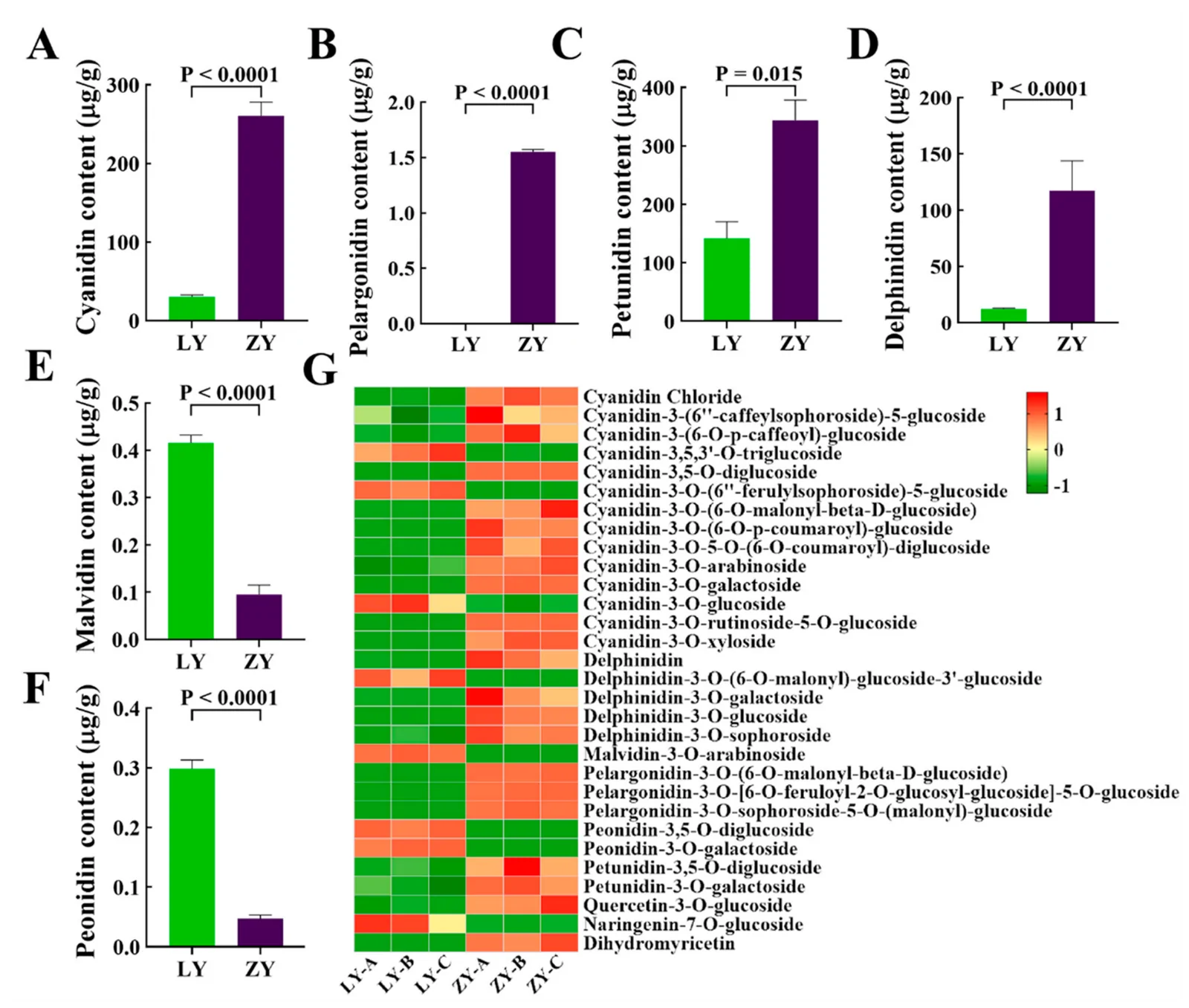
Identification and quantification of anthocyanin-related metabolites in leaves of green leaf (LY) and purple leaf (ZY) mustard by UPLC-MS/MS. (**A**–**F**) Contents of six anthocyanin aglycones: cyanidin (**A**), pelargonidin (**B**), petunidin (**C**), delphinidin (**D**), malvidin (**E**), and peonidin (**F**) in LY and ZY leaves. (**G**) Heatmap showing the relative abundance of 30 identified anthocyanin-related metabolites. Data are presented as means ± standard errors. *p*-values were calculated by Student’s *t*-test.

The anthocyanin types and concentrations in ZY and LY leaves were identified and quantified using UPLC-MS/MS. A total of 30 anthocyanin-related metabolites were identified. Cyanidin, petunidin, and delphinidin were significantly more abundant in ZY and accounted for over 96% of the total anthocyanin content, representing the predominant anthocyanins (Figure 2A,C,D). In contrast, pelargonidin, malvidin, and peonidin were present at trace levels in both ZY and LY (Figure 2B,E,F). To further profile the anthocyanin-related metabolites, a heatmap was constructed to visualize the relative abundance of the identified compounds (Figure 2G). Consistent with the quantitative results for anthocyanin monomers, most cyanidin-, delphinidin-, and petunidin-derived glycosides exhibited higher levels in ZY samples (Figure 2G). Meanwhile, malvidin, peonidin and their derivatives, as well as several flavanol and flavanone compounds, were enriched in LY (Figure 2G). These results indicated that ZY leaves mainly accumulated cyanidin, petunidin, and delphinidin, which contributed to the purple pigmentation, suggesting a divergent anthocyanin biosynthesis pattern between LY and ZY.

### 2.2. Map-Based Cloning of the Candidate Gene Controlling Purple Leaf in Mustard

To determine the inheritance of purple leaf color, we generated F_1_, F_2_, and BC_1_ populations from the LY × ZY cross. All 30 F_1_ individuals had purple leaves, confirming the dominance of purple over green (Table 1). The F_2_ population segregated 668 purple:194 green individuals, fitting a 3:1 ratio (χ^2^ = 2.86, *p* > 0.05). The BC_1_ population (F_1_ × LY) segregated 79 purple:65 green individuals, consistent with 1:1 (χ^2^ = 1.36, *p* > 0.05). These segregation patterns indicate that a single dominant gene controls purple leaf color in *B. juncea*, designated *BjZY*.

**Figure 3:**
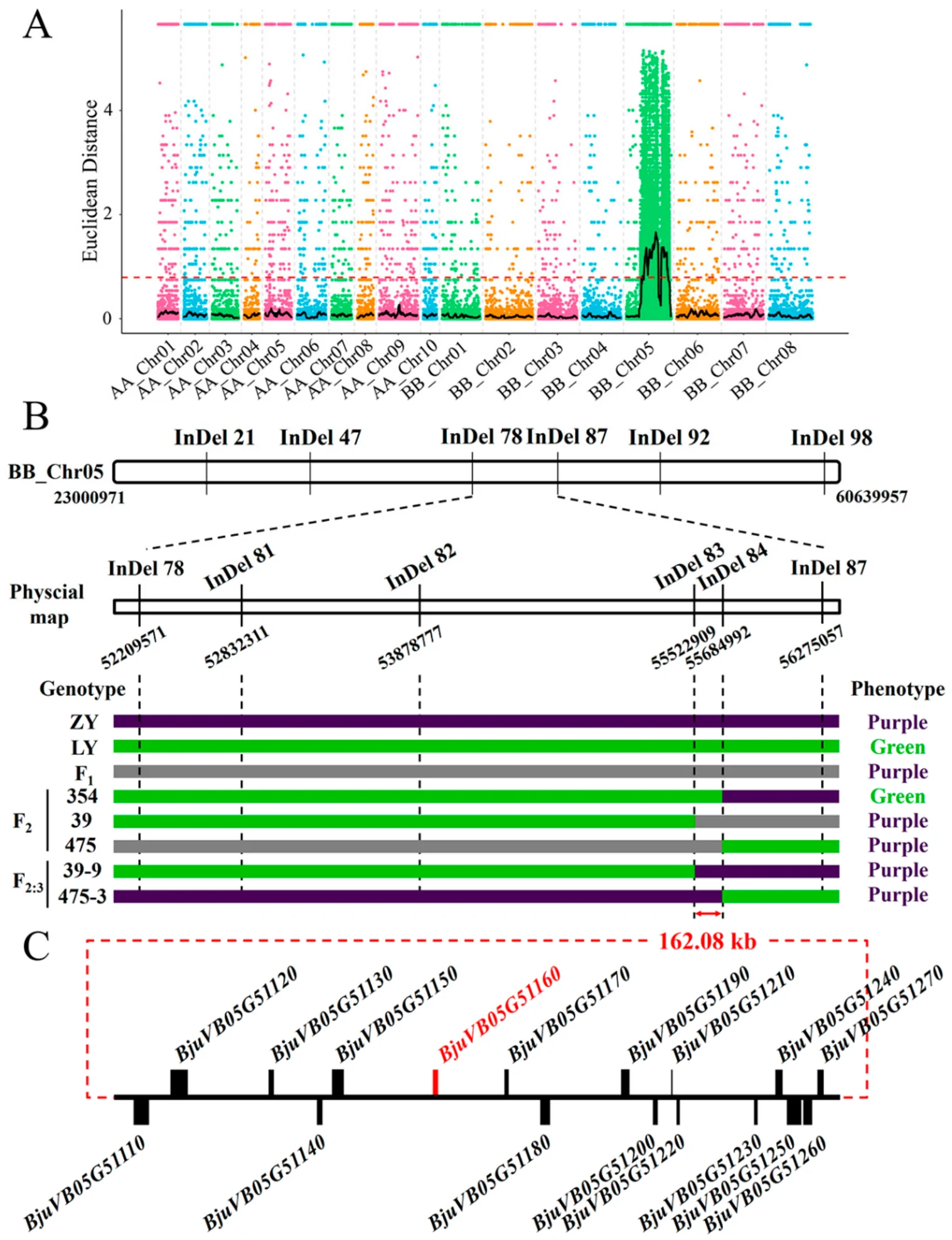
Bulk segregant analysis (BSA-seq) and fine mapping of the *BjZY* locus controlling purple leaf color. (**A**) Euclidean distance plot from BSA-seq showing the candidate genomic region associated with purple leaf trait on BB_Chr05. The red dashed line indicates the significance threshold. (**B**) Fine-mapping of *BjZY* using InDel markers in the F_2_ and F_2:3_ segregating population. (**C**) Gene annotation within the 162.08 kb candidate region. The red rectangle highlights the candidate R2R3-MYB gene *BjuVB05G51160*.

BSA-seq was performed for the preliminary mapping of the *BjZY* locus. After filtering, 269,103,000 and 222,051,146 clean reads were obtained from LY and ZY DNA bulks, respectively (Table S2). More than 98.8% of the raw reads were mapped to the *B. juncea* reference genome T84-66.V2.0, and a total of 1,862,974 SNPs and 582,946 InDels were identified between the two bulks (Table S2). Euclidean distance analysis identified the candidate region on chromosome BB_Chr05 associated with purple leaf (Figure 3A). To further narrow the target region, we developed InDel markers across the BSA-seq candidate interval on BB_Chr05 and conducted three rounds of fine mapping with progressively larger F_2_ populations (Figure 3B). First, 1 ZY, 1 LY, 1 F_1_, and 93 F_2_ individuals were genotyped with 20 polymorphic InDel markers spanning the BSA-seq interval, which preliminarily placed the *BjZY* locus between InDel-78 (52,209,571 bp) and InDel-87 (56,275,057 bp). Additional markers (Table S1) were then developed within this interval and genotyped in 862 F_2_ individuals. A total of 268 F_2:3_ individuals derived from two F_2_ heterozygous recombinants (39 and 475) were used for phenotypic and genotypic validation. Finally, the *BjZY* locus was narrowed to a 162.08 kb region on BB_Chr05 flanked by InDel-83 (55,522,909 bp) and InDel-84 (55,586,957 bp; Figure 3B). According to the reference genome, 17 genes were annotated in this region. Among them, *BjuVB05G51130* and *BjuVB05G51160* encode two R2R3-MYB transcription factors related to anthocyanin biosynthesis (Table S3).

### 2.3. Promoter Variants of BjMYB113 Underlie the Purple-Leaf Trait in Mustard

**Figure 4:**
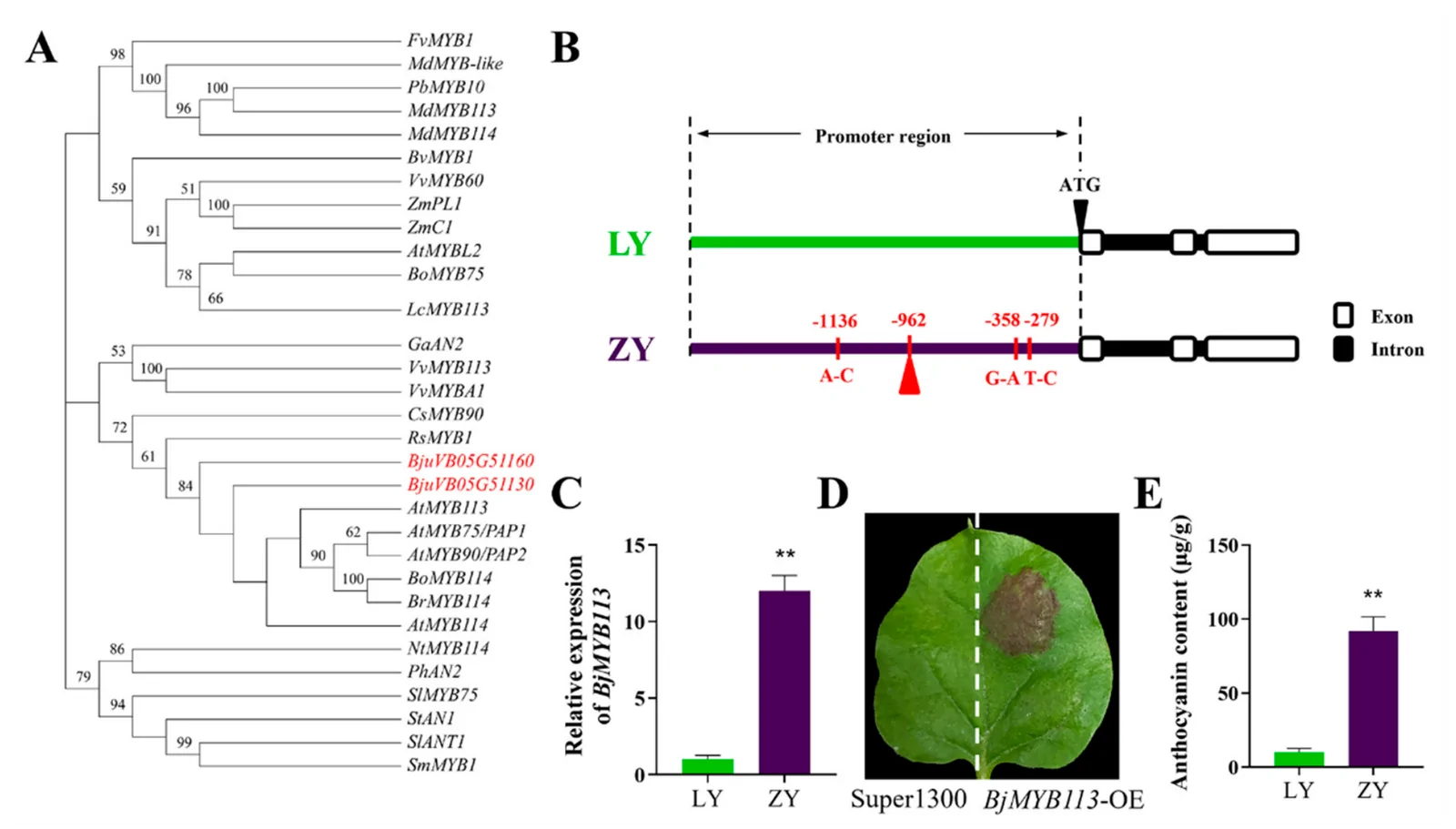
Phylogenetic analysis, sequence variation, expression and functional validation of the candidate R2R3-MYB gene *BjMYB113* (*BjuVB05G51160*). (**A**) Phylogenetic tree of *BjuVB05G51130*, *BjuVB05G51160*, and known anthocyanin-regulating R2R3-MYB proteins from different plant species. (**B**) Schematic diagram of sequence variations in the promoter region of *BjMYB113* between LY and ZY. Red vertical lines indicate SNP sites. (**C**) Relative expression level of *BjMYB113* in LY and ZY leaves. (**D**) Leaf phenotype of tobacco transiently overexpressing *BjMYB113* (BjMYB113-OE); Super1300 served as an empty vector control. (**E**) Anthocyanin content in tobacco leaves with transient overexpression of *BjMYB113*. Values represent means ± SEs. ** indicates significant difference at *p* < 0.01 by Student’s *t*-test.

To confirm the strongest candidate gene, a phylogenetic tree was constructed using *BjuVB05G51130* and *BjuVB05G51160* (*BjMYB113*), characterizing R2R3-MYB genes from *Arabidopsis*, *Brassica*, and other species (Figure 4A). The results showed that both *BjuVB05G51130* and *BjuVB05G51160* were orthologous to MYB genes that have been reported to regulate anthocyanin biosynthesis in *Arabidopsis* (Figure 4A). Sequencing analysis revealed that no difference was found in the *BjuVB05G51130* promoter or CDS between the two parents (Figure S1). However, three SNPs and a 25 bp insert in the *BjuVB05G51160* promoter were detected in ZY compared with LY (Figure S2). The expression level of *BjMYB113* was 16-fold higher in ZY than in LY, showing a significant difference. These results suggest that variants in the *BjMYB113* promoter are likely associated with its higher expression in ZY. The transient expression of *BjMYB113* in tobacco leaves induced purple pigmentation, confirming its function in anthocyanin biosynthesis and accumulation. These findings suggest that promoter variants of *BjMYB113* are likely associated with its altered expression and the consequent anthocyanin accumulation in ZY leaves.

### 2.4. Transcriptome Profiling Reveals the Regulatory Network of BjMYB113 in Leaf Coloration

**Figure 5:**
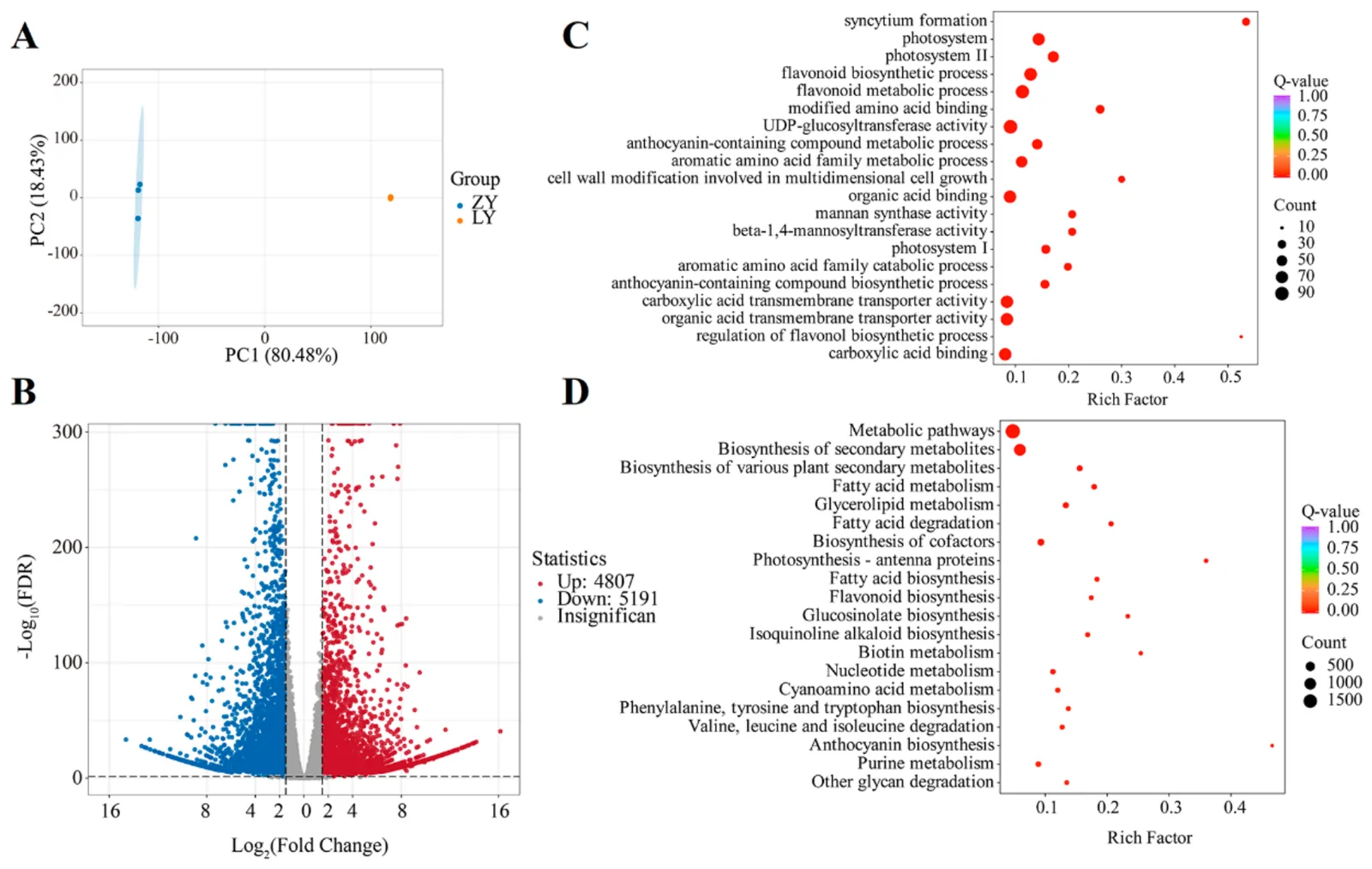
Transcriptome profiling and enrichment analysis between ZY and LY leaves. (**A**) Principal component analysis (PCA) of transcriptome data from ZY and LY leaf samples. (**B**) Volcano plot showing differentially expressed genes (DEGs) between ZY and LY. Red dots represent upregulated DEGs, blue dots represent downregulated DEGs, and gray dots indicate genes with no significant expression change (DEG threshold: |log_2_FC| ≥ 1.5, *p* < 0.05). (**C**) GO enrichment bubble plot of DEGs. (**D**) KEGG enrichment bubble plot of DEGs. The color of the dots represents the Q-value (adjusted *p*-value for enrichment test) and the dot size indicates the gene count enriched in each term.

To unravel the potential network of *BjMYB113* involved in regulating leaf coloration, transcriptomic analysis was performed using ZY and LY leaves at 45 d after sowing. Six RNA-seq libraries were constructed and sequenced. Approximately 44.17 G clean bases were generated, with Q30 values above 97%. The GC content ranged from 46.9% to 47.84%, and the mapping rates ranged from 90.46% to 91.07% across samples (Table S4). Principal component analysis (PCA) showed that the samples of ZY and LY were distinctly separated (Figure 5A). After filtering by |log_2_FC| > 1.5 and *p* < 0.05, a total of 9998 DEGs were identified between ZY and LY, 4807 of which were upregulated and 5191 were downregulated (Figure 5B). GO enrichment analysis showed that terms associated with flavonoid metabolic process (87 DEGs), flavonoid biosynthetic process (78 DEGs), anthocyanin-containing compound metabolic (48 DEGs), and anthocyanin-containing compound biosynthetic processes (34 DEGs) were highly enriched in the biological process category (Figure 5C). KEGG enrichment analysis identified metabolic pathways and biosynthesis of secondary metabolites as the top enriched terms (Figure 5D). Notably, anthocyanin biosynthesis exhibited the largest rich factor, indicating that this pathway was most strongly activated and contributed substantially to the purple leaf phenotype (Figure 5D). Interestingly, photosynthesis-related pathways were enriched in both GO and KEGG analyses, consistent with the decrease in chlorophyll content in ZY, implying that the high expression of *BjMYB113* and the over-accumulation of anthocyanins adversely impact photosynthesis. Overall, GO and KEGG enrichment results indicated that the purple leaf phenotype of *B. juncea* was mainly attributed to the reprogramming of flavonoid and anthocyanin metabolism.

**Figure 6:**
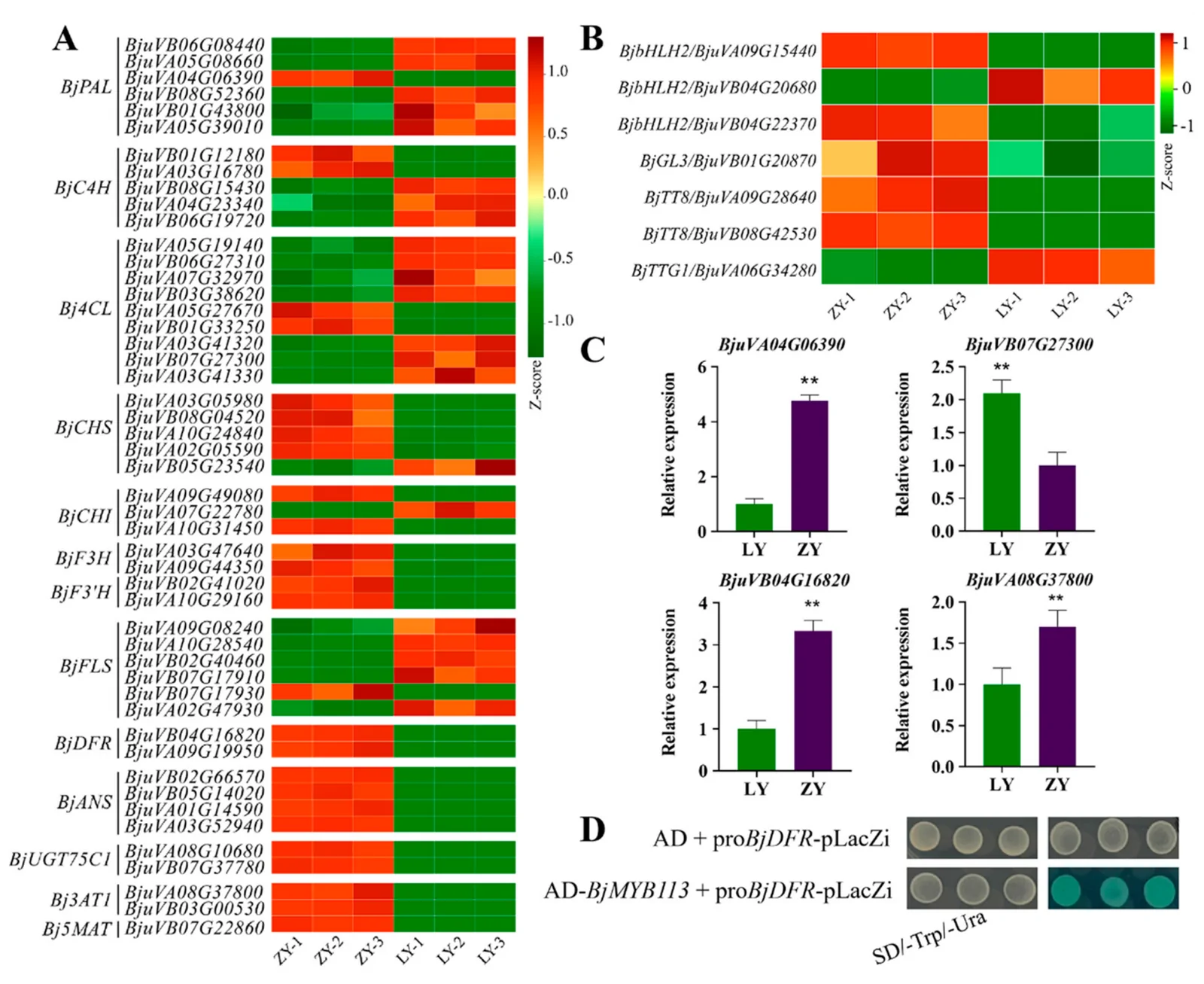
Expression profiles of anthocyanin pathway genes and verification of the direct interaction between *BjMYB113* and the *BjDFR* promoter. (**A**) Heatmap showing the expression patterns of flavonoid and anthocyanin biosynthetic structural genes. Expression values were normalized by Z-score. (**B**) Heatmap showing expression of MBW complex component genes. (**C**) qRT-PCR validation of selected differentially expressed structural genes. Data are means ± SDs (n = 3). ** indicates significant difference at *p* < 0.01 by Student’s *t*-test. (**D**) Yeast one-hybrid (Y1H) assay verifying that *BjMYB113* binds to the promoter of *BjDFR*.

To dissect the transcriptional basis underlying flavonoid and anthocyanin biosynthesis, we profiled the expression of related genes and their upstream regulators using Z-score normalized RNA-seq data (Figure 6A). The early biosynthetic genes (*BjPAL*, *BjC4H*, *Bj4CL*, and *BjCHS*) exhibited a divergent expression pattern, with some preferentially transcribed in LY and others such as BjPAL expressed at higher levels in ZY. In contrast, all the late biosynthetic genes (*BjDFR*, *BjANS*, *BjUGT75C1*, *Bj3AT1*, *Bj5MAT*) were highly expressed in ZY but were strongly suppressed in LY (Figure 6A). The results suggest that gene expression was preferentially directed toward anthocyanin biosynthesis in ZY, whereas the flavonoid branch was favored in LY. *BjbHLH2*, *BjTT8*, and *BjGL3*, components of the MYB-bHLH-WD40 (MBW) complex, were also highly upregulated in ZY, paralleling the expression of *BjMYB113* and structural genes (Figure 6B). The expression of the four above genes was further confirmed by RT-qPCR analysis. The results showed that the expression levels of *BjPAL* (*BjuVA04G06390*), *BjDFR* (*BjuVB04G16820*), and *Bj3AT1* (*BjuVA08G37800*) were significantly higher in ZY than in LY, whereas *Bj4CL* (*BjuVB07G27300*) exhibited the opposite pattern (Figure 6C). These results further validated the reliability of the transcriptome data. We performed Y1H assays to detect whether *BjMYB113* directly regulates anthocyanin structural genes. The co-transformation of *AD-BjMYB113* with pro*BjDFR*-pLacZi produced blue colonies on SD/-Trp/-Ura-X-Gal selective medium (Figure 6D), indicating that *BjMYB113* might directly activate the *BjDFR* promoter. The upregulation of *BjANS* and *BjUGT75C1* may result from either direct binding by BjMYB113 or indirect regulation mediated by bHLH components of the MBW complex.

## 3. Discussion

Anthocyanins play crucial roles in plant coloration, stress tolerance and health benefits, and are derived from six major aglycone classes based on their aglycones, including cyanidin, delphinidin, pelargonidin, petunidin, peonidin, and malvidin [36,37]. The glycosylation and acylation patterns of anthocyanin compounds determine the hue and stability of red-to-purple pigmentation, resulting in species- and variety-specific anthocyanin profiles [38]. In Brassicaceae, purple vegetables, such as Chinese cabbage [29], cauliflower [32], rapeseed [31], and pak choi [39], predominantly accumulate cyanidin-type glycosides, a profile largely determined by flavonoid 3′-hydroxylase (F3′H), which channels phenylpropanoid flux towards the cyanidin branch [40]. In our materials, the anthocyanin content reached 0.79 mg/g in purple ZY leaves compared to 0.18 mg/g in green LY leaves, and cyanidin, petunidin, and delphinidin together accounted for more than 96% of the total anthocyanins (Figure 2). The dominance of cyanidin is fully consistent with the profiles reported for other purple *Brassica* vegetables [33]. However, the notable share of petunidin and delphinidin in ZY is less commonly seen in *Brassica* vegetables. For example, petunidin and delphinidin were absent or present only in trace amounts in pak choi, cauliflower, and cabbage [33,39,41]. Previous studies also reported that a high level of delphinidin was detected in the purple-leaf mustard carrying the *BjMYB113a* allele, but the *BjMYB113* introgression lines in *B. napus* with purple leaf do not accumulate petunidin or delphinidin [31,42]. This suggests that the abnormal petunidin and delphinidin accumulation profiles in mustard, distinct from those of *Brassica* vegetables, may be species-specific and independent of *BjMYB113*.

*MYB113* and homologs function in an MBW ternary complex with bHLH and WD40 partners to regulate anthocyanin biosynthesis in plants, such as *Arabidopsis* [17], tomato [43], apple [44], cauliflower [32], cabbage [45], tobacco [46,47], grape [48], and litchi [49]. In this study, the *BjZY* locus was mapped in the 162.08 kb interval on chromosome BB_Chr05 (Figure 3). In the interval, two MYB transcription factors, *BjuVB05G51130* and *BjuVB05G51160*, are annotated and clustered together with the *Arabidopsis PAP1*, *PAP2*, *MYB113*, and *MYB114* (Figure 4). *BjuVB05G51130* shows no variants in either the promoter region or the gene sequence (Figure S1). Thus, *BjuVB05G51160*, named *BjMYB113*, is the strongest candidate gene with a predicted role in the transcriptional regulation of anthocyanin metabolism. The function of *BjPur* (*BjMYB113*) is confirmed via CRISPR/Cas9-mediated gene knockout in mustard [50]. In *B. juncea*, *BjMYB113* harbors a wide range of natural genetic variants, including a 1268 bp SINE transposon insertion in the first intron, 45 SNPs and 8 InDels in the promoter, and an 1033 bp insertion in the 3′ region [42,51]. We identified 3 SNPs and a 25 bp insertion in the *BjMYB113* promoter region, which are completely different from the variants previously reported. These promoter polymorphisms are associated with altered gene expression, while the coding sequence of *BjMYB113* remains unchanged. The purple leaf allele drives high-level transcription of *BjMYB113*, whereas the green leaf allele confers 16-fold-lower transcription, indicating a quantitative rather than qualitative change. In cauliflower, the activation of the *BoMYB2* gene is caused by a point mutation and/or a 1 bp insertion in its promoter region, which is distinct from the 7606 bp CACTA-like transposon insertion in the promoter of purple kale and kohlrabi. In purple pak choi, two short fragment insertions in the promoter region of *BraANS.A03* in green-leaf material likely interfere with normal gene expression, blocking anthocyanin biosynthesis and resulting in a green leaf phenotype [52]. Together with previous studies, these findings add to a growing body of evidence that cis-regulatory evolution is an important driver of anthocyanin accumulation and provide many useful variant sites for improving leaf color traits.

Our RNA-seq data identified 9998 DEGs between purple and green leaves (4807 upregulated, 5191 downregulated), providing a transcriptome-wide view of this regulatory rewiring. GO and KEGG enrichment analyses confirmed that anthocyanin biosynthesis was the most strongly activated pathway, while photosynthesis-related pathways were suppressed, consistent with the significantly lower chlorophyll content in purple leaves. This trade-off between anthocyanin accumulation and photosynthetic capacity likely reflects resource competition between the phenylpropanoid and chlorophyll pathways. A notable finding from our transcriptomic analysis is the co-upregulation of the MBW complex components *BjbHLH2*, *BjTT8*, and *BjGL3* alongside *BjMYB113* in purple leaves. This co-expression pattern is consistent with a feed-forward regulatory loop. In *Arabidopsis*, *MYB113* functions as a component of the MBW complex with *GL3*/*EGL3* and *TTG1* to directly activate late biosynthetic genes, and its activity is *TTG1*- and *bHLH*-dependent [17]. This implies that *BjMYB113* may function together with *BjbHLH2*, *BjTT8*, and *BjGL3* by forming an MBW complex to regulate anthocyanin biosynthetic genes. The late biosynthetic genes *BjDFR*, *BjANS*, *BjUGT75C1*, *Bj3AT1*, and *Bj5MAT* were strongly upregulated in purple leaves, while early biosynthetic genes including *BjPAL*, *BjC4H*, *Bj4CL*, and *BjCHS* were preferentially expressed in green leaves. Consistently, *BrMYB2* expression similarly accompanies the upregulation of *BrTT8*, *BrF3′H*, *BrDFR1*, and *BrANS1* in *B. rapa* [29]. Y1H assays prove that *BjMYB113* regulates *BjDFR*’s expression by directly binding to its promoter, but additional target promoters remain to be tested. The divergent expression pattern indicates that *BjMYB113* specifically activates the late anthocyanin branch without broadly inducing the entire flavonoid pathway.

## 4. Materials and Methods

### 4.1. Plant Materials

Two *B. juncea* inbred lines with contrasting leaf colors were used in this study. F_1_ plants were generated by crossing the purple leaf ZY (Zijinshuicai) as the male parent with the green leaf LY (Lvhuayejie) as the female parent. These two inbred lines are preserved at the Institute of Vegetables, Anhui Academy of Agricultural Sciences, and their phenotypic authenticity has been maintained by successive selfing. The F_2_ population was derived from the self-pollination of F_1_ individuals. The BC_1_ population was produced by backcrossing F_1_ plants to LY. All plants were grown in the experimental field of the Gangji Ecological Agriculture Experimental and Demonstration Base, Anhui Academy of Agricultural Sciences from September 2024 to May 2025. Leaf color phenotypes were monitored at the seedling stage (45 d after sowing) and confirmed at the rosette stage.

### 4.2. Plant Pigment Content Measurement

Anthocyanins were extracted from 0.5 g of fresh leaves of LY and ZY by vortexing samples with steel beads and 1 mL of methanol/water (7:3, *v*/*v*, and 0.1% formic acid) for 30 s, followed by homogenization at 45 Hz for 15 min and sonication in an ice bath for 30 min. After centrifugation (12,000 rpm, 4 °C, 15 min), the supernatant was filtered (0.22 μm) for analysis. Chromatographic separation was performed on a Waters ACQUITY I-Class UHPLC (Waters Corporation, Milford, MA, USA). MS detection was performed using a SCIEX QTRAP 6500+ (SCIEX, Framingham, MA, USA) triple quadrupole in multiple reaction monitoring (MRM) mode. Metabolites were identified achieved by matching the precursor ion, diagnostic MS/MS fragments and retention time with authentic standards. The detected analytes were intact anthocyanin glycosides. Calibration curves were constructed with authentic anthocyanidin aglycone standards, and glycoside contents were mathematically converted to aglycone equivalents by molecular weight calculation. Three biological replicates and three technical replicates were measured for each line.

Fresh leaf tissues (0.1 g, avoiding the midrib) were used to determine the total carotenoids, total chlorophyll, total flavonoids and total anthocyanins. The carotenoid, chlorophyll and flavonoid contents were measured using Solarbio assay kits (BC4335, BC0995 and BC1330, respectively; Beijing, China), while total anthocyanins were extracted with 1% (*v*/*v*) HCl methanol at 4 °C in darkness for 24 h. After centrifugation, the absorbance of the supernatant was determined at 530 nm by UV spectrophotometry using 1% (*v*/*v*) HCl-methanol as the blank.

### 4.3. Bulk Segregant Analysis Sequencing (BSA-Seq)

Genomic DNA was extracted from young leaves of 30 purple leaf and 30 green leaf F_2_ individuals using a CTAB method. Equal quantities of DNA from each set of 30 individuals were pooled to construct purple and green bulks. Paired-end libraries were constructed from bulk DNA and sequenced on the Illumina NovaSeq platform (Biomarker Technologies Co., Ltd., Beijing, China) to approximately 40x depth for each bulk. Clean reads were aligned to the *B. juncea* reference genome T84-66.V2.0 using BWA-MEM2 [53]. SNP calling was performed with GATK [54]. The Euclidean distance (ED) was calculated for each SNP locus based on allele frequencies in the purple and green bulks. Sliding window analysis was then performed to identify candidate intervals with elevated ED values.

### 4.4. InDel Marker Development and Fine Mapping

InDel polymorphisms in the BSA-seq candidate interval on BB_Chr05 were identified from resequencing data. Primers flanking each InDel were designed using Primer3 (https://bioinfo.ut.ee/primer3-0.4.0/, accessed on 12 October 2026). The produced InDel markers were confirmed to be polymorphic by genotyping the ZY line, LY line, and F_1_ population (Table S1). The amplified PCR products were genotyped on 12% denaturing polyacrylamide gels or 2% agarose gels, depending on the extent of polymorphism.

Gene mapping was conducted using progressively larger F_2_ populations. First, 1 ZY, 1 LY, 1 F_1_, and 93 F_2_ individuals were genotyped with all InDel markers spanning the BSA-seq interval. Then, the population was expanded to 862 F_2_ individuals and genotyped with key markers. In the end, additional markers between the closest flanking markers were developed and genotyped. Recombinant individuals within the interval were identified, and their phenotypes and genotypes were recorded to pinpoint crossover breakpoints. Two heterozygous F_2_ recombinants (39 and 475) were self-pollinated to develop F_2_:_3_ families (268 individuals) for the phenotypic and genotypic validation of the mapping interval. The physical size of the candidate interval was calculated from marker positions on the reference genome T84-66.V2.0.

### 4.5. Candidate Gene Prediction and Phylogenetic Analysis

Annotated genes within the fine-mapped interval were retrieved from the *B. juncea* genome annotation (Table S2). Sequences were searched against the NCBI database using BLASTP (https://blast.ncbi.nlm.nih.gov, accessed on 12 October 2026). Protein domains were predicted using InterProScan v5.63-95.0 (European Bioinformatics Institute, Hinxton, Cambridgeshire, UK; https://www.ebi.ac.uk/interpro/, accessed on 12 October 2026) and the Pfam database (http://pfam.xfam.org/, accessed on 12 October 2026). Genes with homology to anthocyanin-related transcription factors were prioritized. The full-length coding sequence of *BjMYB113* was amplified from ZY leaf cDNA (Table S1). The predicted BjMYB113 protein was aligned with R2R3-MYB proteins from *Fragaria vesca* (FvMYB1, ABX79948.1), *Malus domestica* (MdMYB113, NP_001315777.1; MdMYB114, NP_001280757.1; MdMYB-like, NP_001280759.1), *Pyrus bretschneideri* (PbMYB10, AGZ15307.1), *Beta vulgaris* (BvMYB1, XP_010665861.1), *Vitis vinifera* (VvMYB113, NP_001268189.1; VvMYBA1, ABL14066.1; VvMYB60, XP_002282589.1), *Zea mays* (ZmC1, NP_001105885.1; ZmPL1, NP_001358856.1), *Arabidopsis thaliana* (AtMYB75/PAP1, NP_176057.1; AtMYB90/PAP2, NP_176813.1; AtMYB113, NP_176811.1; AtMYB114, NP_176812.1; AtMYBL2, CAO7032889.1), *Brassica oleracea* (BoMYB114, WEY29604.1; BoMYB75, XP_013590777.1), *Litchi chinensis* (LcMYBx, UCU01159.1), *Gossypium arboreum* (GaAN2, AHA59265.1), *Citrus sinensis* (CsMYB, NP_001275818.1), *Raphanus sativus* (RsMYB1, QYJ10361.1), *Brassica rapa* (BrMYB114, WEY29609.1), *Nicotiana tabacum* (NtMYB114, NP_001312447.1), *Petunia hybrida* (PhAN2, WJN62204.1), *Solanum lycopersicum* (SlANT1, YCN21065.1; SlMYB75, NP_001265992.1), *Solanum tuberosum* (StAN1, NP_001275311.1), and *Solanum melongena* (SmMYB1, QKV27976.1). A neighbor-joining phylogenetic tree was constructed using MEGA11 (https://www.megasoftware.net/, accessed on 12 October 2026) with the Poisson model and 1000 bootstrap replicates.

### 4.6. RNA Extraction, RT-qPCR, and RNA-Seq Analysis

Total RNA was extracted from seedling-stage leaves harvested at 45 d after sowing at 10:00 a.m. using the RNAprep Pure Plant Plus Kit (Tiangen, Beijing, China) according to the manufacturer’s protocol. First-strand cDNA was synthesized from 1.5 µg of total RNA using the PrimeScript RT reagent Kit with gDNA Eraser (Takara, Dalian, China). Quantitative PCR was performed on a CFX96 Touch Real-Time PCR Detection System (Bio-Rad, Hercules, CA, USA) using TB Green Premix Ex Taq II (Takara, Dalian, China). *BjEF-1-α* was used as the internal control. Reactions for the reference genes were included in each plate. Three biological replicates with three technical replicates were assayed for each sample. The relative expression was calculated using the 2^−ΔΔCt^ method [55].

RNA-seq libraries were constructed from LY and ZY fresh leaves at 45 d after sowing with three replicates and sequenced on the MGI sequencing platform by Metware Biotechnology Co., Ltd. (Wuhan, China). Clean reads were mapped to the *B. juncea* reference genome using HISAT2 [56]. Gene expression was quantified as FPKM using StringTie [57]. Differentially expressed genes (DEGs) were identified using DESeq2 with criteria |log_2_FC| ≥ 1.5 and *p* < 0.05. GO and KEGG enrichment analyses of DEGs were performed using cluster Profiler against the Gene Ontology (https://geneontology.org/) and KEGG databases (https://www.kegg.jp/). Heatmaps of Z-score normalized FPKM values for anthocyanin and flavonoid pathway genes were generated with the pheatmap package in R (https://www.r-project.org/).

### 4.7. Yeast One-Hybrid (Y1H) Assay

The full-length *BjMYB113* CDS and *BjDFR* promoter were cloned into the pGADT7-AD vector and pLacZi reporter vector, respectively (Table S1). The AD-*BjMYB113* fusion and pro*BjDFR*-pLacZi reporter were co-transformed into yeast strain EGY48 using the lithium acetate method. Transformants were selected on SD/-Trp/-Ura medium. Protein-DNA interactions were assessed by growth on SD/-Trp/-Ura/X-Gal medium with 2% raffinose and 2% galactose at 30 °C for 3–5 d. Empty AD co-transformed with pro*BjDFR*-pLacZi served as the negative control. Blue colony formation indicated positive interaction.

### 4.8. Transient Expression in Tobacco Leaf

The full-length *BjMYB113* CDS without the stop codon was cloned downstream of the CaMV35S promoter in the Super1300 vector (Table S1). The construct was introduced into *Agrobacterium tumefaciens* strain GV3101 by heat-shock transformation. Agrobacterium cells were cultured in LB liquid medium containing kanamycin and rifampicin at 28 °C. When the culture reached an optical density at 600 nm (OD600) of about 0.8, the cells were harvested and resuspended in infiltration buffer (10 mM MES, pH 5.8, 10 mM MgCl_2_, 200 μM acetosyringone). The suspension was infiltrated into tobacco (*Nicotiana tabacum*) leaves using a 1 mL needleless syringe. The empty Super1300 vector was infiltrated as a negative control. The infiltrated plants were maintained under a 16 h light/8 h dark photoperiod at 22–25 °C. Phenotypic observation and sampling were performed at 5 days after infiltration, and the total anthocyanin content was determined as described in Section 4.2.

### 4.9. Statistical Analysis

Statistical analysis was performed using SPSS software 22.0 (IBM, Armonk, NY, USA; https://www.ibm.com/cn-zh/products/spss, accessed on 12 October 2026). Differences between groups were assessed by two-tailed unpaired Student’s *t*-tests. *, *p* < 0.05 and **, *p* < 0.01 were considered statistically significant.

## 5. Conclusions

In conclusion, we identify an R2R3-MYB transcription factor, *BjMYB113*, as the candidate gene underlying purple leaf trait on chromosome BB_Chr05 in *B. juncea*. Variants (three SNPs and a 25 bp insertion) in the *BjMYB113* promoter alter its expression, leading to the leaf color change. High expression of *BjMYB113* triggers metabolic flux toward flavonoid and anthocyanin biosynthesis by regulating structural genes such as *BjDFR*, thereby promoting anthocyanin biosynthesis and accumulation and leading to leaf pigmentation. These findings provide a genetic and molecular foundation for the marker-assisted breeding and metabolic engineering of anthocyanin content in *B. juncea*.

## Figures and Tables

**Table 1:** Segregation of leaf color in populations derived from the cross LY (green) × ZY (purple).

Population	Purple Plants	Green Plants	Total	Expected Ratio	Expected (Purple:Green)	χ^2^	*p*-Value
LY	15	0	15				
ZY	0	15	15				
F_1_	30	0	30				
F_2_	668	194	862	3:1	646.5:215.5	2.86	0.09
BC1(F_1_ × LY)	79	65	144	1:1	72:72	1.36	0.24

## Data Availability

All data generated or analyzed during this study are included in this published article. The raw RNA-seq data (accession: PRJCA076713) and BSA-seq data (accession: PRJCA078162) have been deposited in the Genome Sequence Archive (GSA) of the China National Center for Bioinformation.

## Supplementary Materials

The following supporting information can be downloaded at https://www.mdpi.com/article/10.3390/plants15213463/s1. Figure S1. Sequence alignment of the promoter and coding sequence of BjuVB05G51130; Figure S2. Sequence alignment of the promoter and coding sequence of BjuVB05G51160; Table S1. Primer sequences used in this study; Table S2. Summary of BSA-seq data quality and mapping statistics; Table S3. Candidate genes within the fine-mapped interval; Table S4. Summary of RNA-seq data quality and mapping statistics.
