# Supplementary material for: Fine-Mapping and Functional Characterization of BjMYB113 in Controlling Purple Leaf Coloration in Brassica juncea
Source: Plants (Basel). 2026 Nov 6;15(21):3463. doi: 10.3390/plants15213463 (PMC13765854; doi:10.3390/plants15213463)
Supplement: Supplementary file 1 [file plants-15-03463-s001.zip › plants-4658306-supplementary.pdf]

Supplementary materials

Fine-mapping and functional characterization of *BjMYB113* in controlling purple leaf coloration in *Brassica juncea*

Zhihuan Ge[1,2,3]*, Mengyi Yu[1,2,4]*, Yinge Xie[1,2,3]*, Haoran Wang[5], Shaojun Ma[1,2,3], Yijian Liu[1,2,3], Shanshan Zhou[6], Xueliang Sun[1,2,3], Feifei Yu[1,2,3#], Mingxia Wang[1,2,3#]

[1]*Key Laboratory of Horticultural Crop Germplasm Innovation and Utilization (Co-Construction by Ministry and Province), Hefei 230011, China*

[2]*Anhui Provincial Key Laboratory for Germplasm Resources Creation and High-Efficiency Cultivation of Horticultural Crops, Hefei 230011, China*

[3]*Institute of Vegetables, Anhui Academy of Agricultural Sciences, Hefei 230011, China*

[4]*Institute of Horticulture, Anhui Academy of Agricultural Sciences, Hefei 230011, China*

[5]*Beijing Vocational College of Agriculture, Beijing 102442, China*

[6]*Jinzhai County Agricultural Industry Development Center, Jinzhai 237300, China*

[#]Corresponding author: Feifei Yu, Mingxia Wang

e-mail: 13965019251@163.com, wangmx302@aaas.org.cn

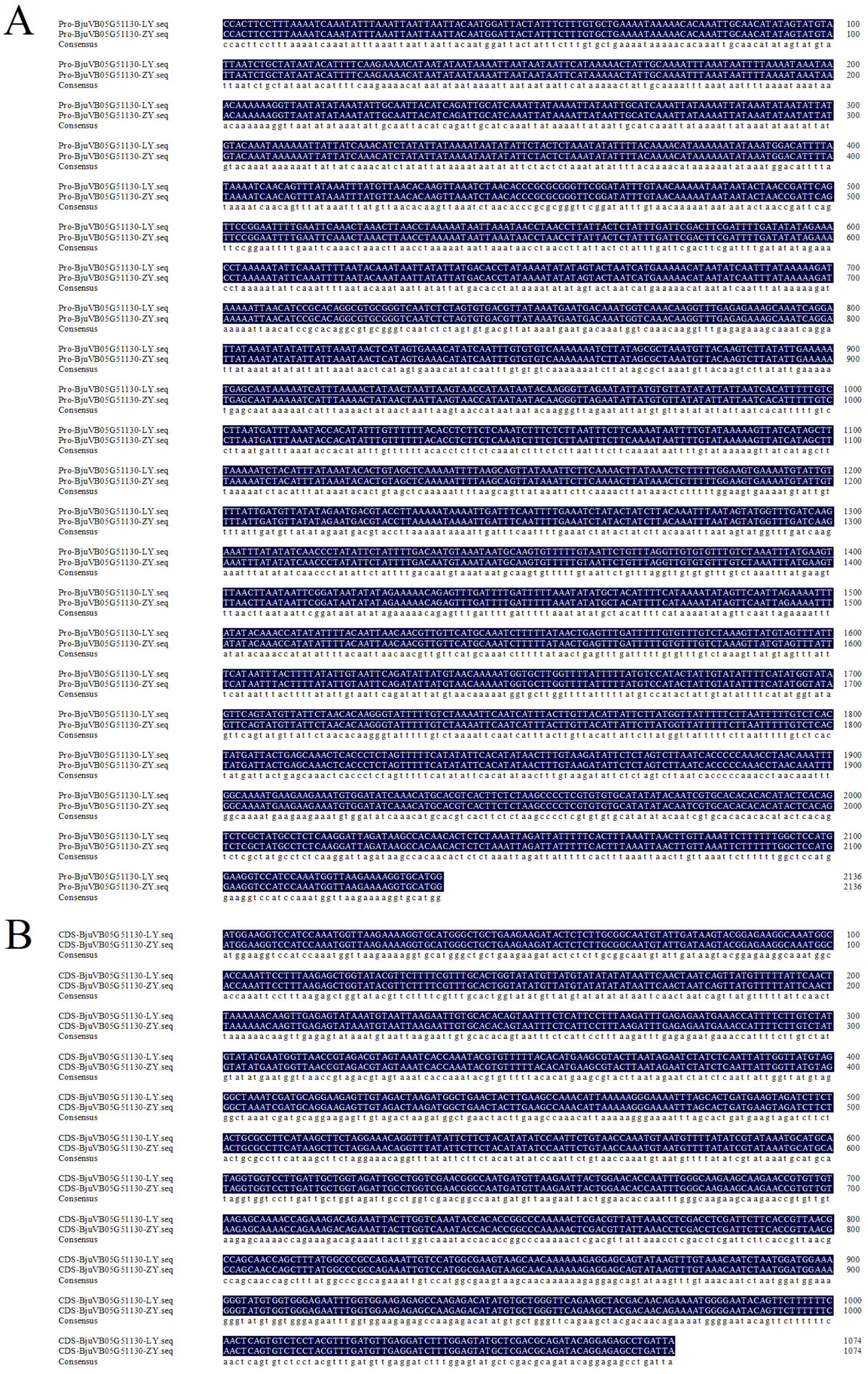

Figure S1. Sequence alignment of the promoter and coding sequence of *BjuVB05G51130*.

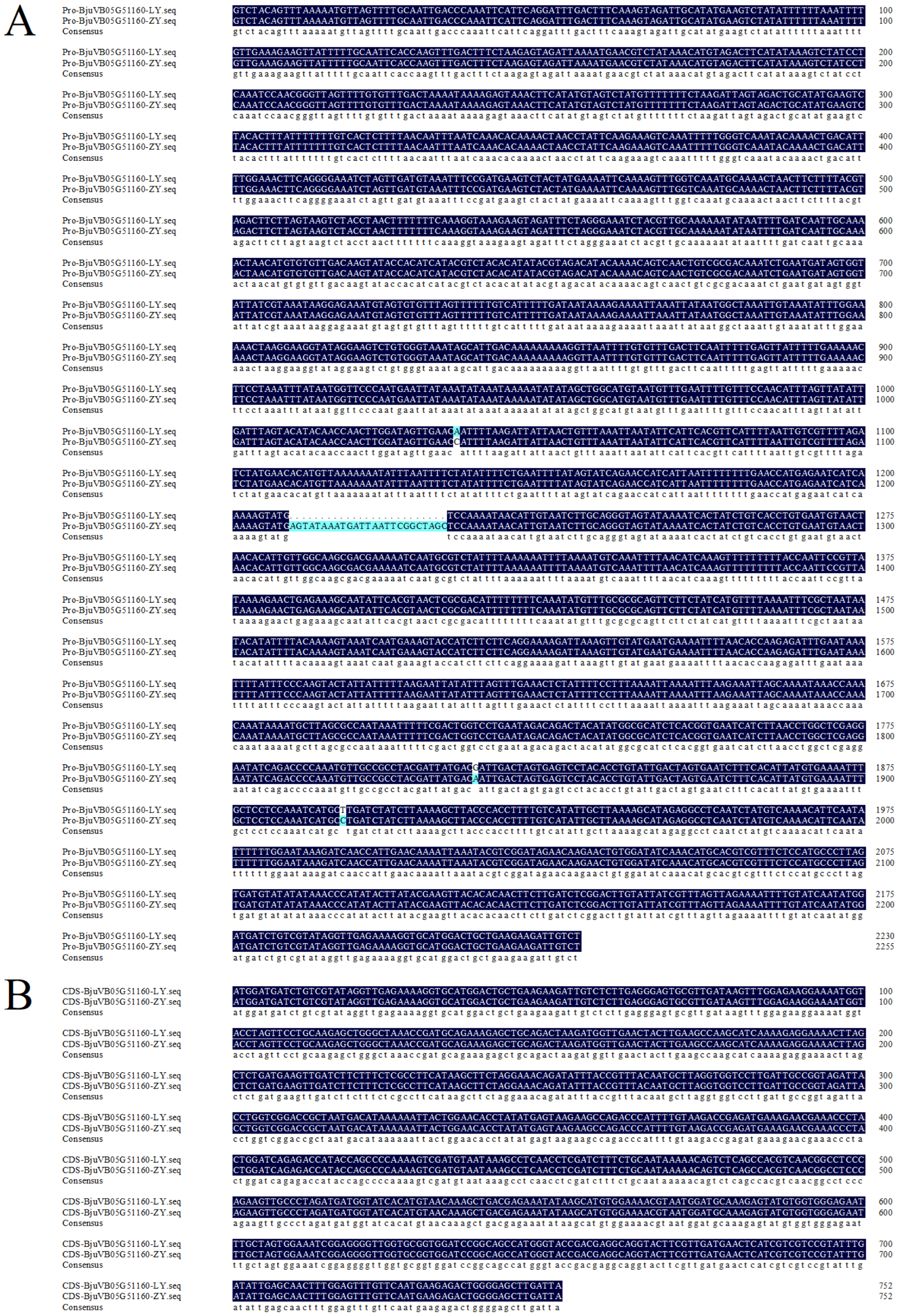

Figure S2. Sequence alignment of the promoter and coding sequence of *BjuVB05G51160*.

Table S1. Primer sequences used in this study.

Marker/gene ID	Forward primer (5'-3')	Reverse primer (5'-3')	Application
InDel21	CTAAAAGAGGTTTGTTTCTACTA	ATGATCCGGACAAGTTGATCT	Mapping
InDel47	GAAAAATTAGAAGTCTGATAAAATT	ATTTGTTTCAACGACTTCGTAAA	Mapping
InDel78	TAAGTTTTTCTCGTATTTGAAATAT	GGATTAATAATTTAACACCAATATT	Mapping
InDel81	AGATTAGCGCATTCAGAGACT	ATGCTTGGTTTTTCTATGCCTT	Mapping
InDel82	TTGTTTGAGAACATCAACTTCAT	TGTTGAAAGTTGATTGGAGAGA	Mapping
InDel83	ACAGAGAAATGATCCAATGATTA	AGTACAGTAGAATAATGGATGAT	Mapping
InDel84	AACCAAACTAAAATCATTTTGTTTT	TCATTTATAGTTGTTTTGTGTTTTA	Mapping
InDel87	GTTAATCGTGATTTATTTTTCTAAA	TGAGCAAGAGATGCTGTTAATT	Mapping
InDel92	TGTGTTGGACTATTAGTCTTTAA	TTCAAATTGTATGACTTTTTAATGA	Mapping
InDel98	TTTCTGAAATTCAAGAACATATCA	GAACACATCGTACTTCCAGAA	Mapping
BjuVB05G51160	ATGGATGATCTGTCGTATAGGTTGAG	ATCAAGCTCCCCAGTCTCTTC	Gene cloning
	CCTCCCAGAAGTTGCCCTAGATG	CACCAACCCCTCCGATTTCCACT	qPCR
BjuVA04G06390	GCAGCGATTGGGTCATGGAGAGT	GGTAGCGTGTGGCATGTCTCCTT	qPCR
BjuVB07G27300	AGACGGAGAAGTACGACCTGAGC	GACTTAGCCACCGTTCCTGACTC	qPCR
BjuVB04G16820	TGATGCGGCTACTGGAACGTGGT	GCGTCATCGTAGCTTCCTTCGTC	qPCR
BjuVA08G37800	CATCCACCACTCTTCCTCTTACC	ATGTTGGAGAGTGGTGGAGAGTG	qPCR
BjuEF1-α	CCCTCCGTCTACCACTTCAG	CACAACCATACCAGGCTTGA	qPCR

Table S2. Summary of BSA-seq data quality and mapping statistics.

Sample	Clean Base	Clean Reads	Mapped (%)	Ave_depth	Q30 (%)	GC (%)	Total SNP number	Total InDel number
LY	40,190,158,297	269,103,000	99.27	42	97.66	36.67	1,862,974	582,946
ZY	33,147,274,938	222,051,146	98.8	34	98.07	36.67		

Table S3. Candidate genes within the fine-mapped interval.

Gene ID	Genome position	Description
BjuVB05G51110	BB_Chr05:55527399..55530809	Probable DNA primase large subunit
BjuVB05G51120	BB_Chr05:55535453..55539409	Replication protein A 70 kDa DNA-binding subunit B
BjuVB05G51130	BB_Chr05:55559411..55560485	Transcription factor MYB75
BjuVB05G51140	BB_Chr05:55568330..55569555	Glutamate dehydrogenase 2
BjuVB05G51150	BB_Chr05:55572211..55574840	1-phosphatidylinositol-3-phosphate 5-kinase FAB1B
BjuVB05G51160	BB_Chr05:55594263..55595446	Transcription factor MYB90
BjuVB05G51170	BB_Chr05:55610229..55611222	Glucuronoxylan 4-O-methyltransferase 1
BjuVB05G51180	BB_Chr05:55618765..55620989	N/A
BjuVB05G51190	BB_Chr05:55636160..55638057	F-box protein
BjuVB05G51200	BB_Chr05:55643392..55644411	N/A
BjuVB05G51210	BB_Chr05:55647539..55647820	N/A
BjuVB05G51220	BB_Chr05:55648660..55649439	REF/SRPP-like protein
BjuVB05G51230	BB_Chr05:55665802..55666678	OBERON-like protein
BjuVB05G51240	BB_Chr05:55670747..55672358	Putative F-box/kelch-repeat protein
BjuVB05G51250	BB_Chr05:55673345..55676613	ELMO domain-containing protein A
BjuVB05G51260	BB_Chr05:55676981..55678952	Probable arabinosyltransferase ARAD1
BjuVB05G51270	BB_Chr05:55680184..55681612	60S ribosomal protein L17-2

Table S4. Summary of RNA-seq data quality and mapping statistics.

Sample	Raw Reads	Raw Base (G)	Clean Reads	Clean Base (G)	Error Rate (%)	Q20 (%)	Q30 (%)	GC Content (%)
LY-1	51,333,368	7.70	50,449,458	7.57	0.01	98.91	97.40	47.65
LY-2	53,535,572	8.03	52,646,204	7.9	0.01	98.94	97.46	47.52
LY-3	51,598,158	7.74	50,471,456	7.57	0.01	99.00	97.62	47.84
ZY-1	43,742,324	6.56	43,002,790	6.45	0.01	98.93	97.46	47.15
ZY-2	47,898,784	7.18	47,258,898	7.09	0.01	98.91	97.40	46.90
ZY-3	51,075,344	7.66	50,585,262	7.59	0.01	98.93	97.45	47.00
